# High-precision inversion of vegetation parameters in the AI era: Integrating hyperspectral remote sensing and deep learning

**DOI:** 10.1016/j.xinn.2025.100868

**Published:** 2025-03-05

**Authors:** Jinkang Hu, Dailiang Peng, Jing M. Chen, Alfredo R. Huete, Le Yu, Zihang Lou, Enhui Cheng, Xuan Yang, Bing Zhang

**Affiliations:** 1 Key Laboratory of Digital Earth Science, Aerospace Information Research Institute, Chinese Academy of Sciences, Beijing 100094, China; 2 International Research Center of Big Data for Sustainable Development Goals, Beijing 100094, China; 3 College of Resource and Environment, University of Chinese Academy of Sciences, Beijing 100094, China; 4 School of Geographical Sciences, Fujian Normal University, Fuzhou 350117, China; 5 Department of Geography and Planning, University of Toronto, Toronto, ON M5S 3G3, Canada; 6 School of Life Sciences, University of Technology Sydney, Ultimo, NSW 2007, Australia; 7 Department of Earth System Science, Ministry of Education Key Laboratory for Earth System Modeling, Institute for Global Change Studies, Tsinghua University, Beijing 100084, China; 8 Institute of Agricultural Remote Sensing and Information Technology Application, College of Environmental and Resource Sciences, Zhejiang University, Hangzhou 310058, China; 9 China Remote Sensing Satellite Ground Station, Aerospace Information Research Institute, Chinese Academy of Sciences, Beijing 100094, China

## Main text

Vegetation traits and parameters serve as key indicators of ecosystem structure, processes, and functioning while also playing crucial roles in biodiversity assessments and the global carbon and water cycles. Remote sensing technologies have emerged as indispensable ecological tools for capturing the spatial and temporal dynamics of vegetation parameters/traits across diverse landscapes and scales. Instead of relying on empirical relationships between remote sensing and vegetation parameters, more sophisticated data models can now be developed that leverage both vegetation spectral and structural signals to account for the complex interactions between radiation and vegetation canopies and provide a more comprehensive and accurate assessment of vegetation parameters. The proliferation of remote sensing data, particularly with the increasing availability of satellite-based imaging spectroscopy, has created an unprecedented dataset of information about the Earth’s terrestrial biosphere. This exponential growth in data, coupled with an increasing demand for more precise vegetation parameter retrievals, has spurred the development of new methodologies aimed at creating efficient, accurate, and adaptable data analysis techniques and applications for deriving vegetation parameters from remote sensing data.^1^

**Figure 1:**
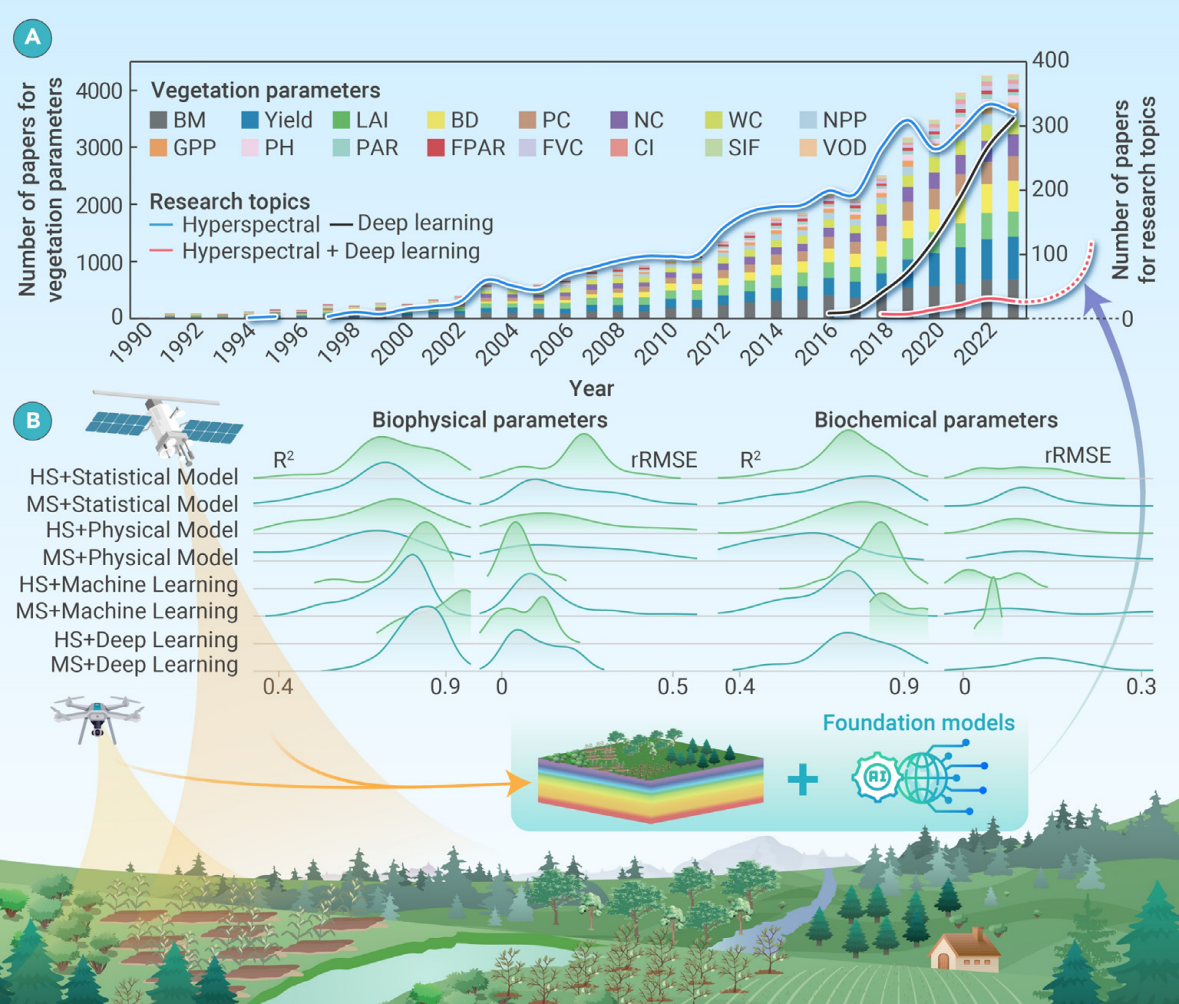
Based on the Core Collection of Web of Science (A) The bar chart illustrates the number of published papers on the remote sensing inversion for various vegetation parameters from 1990 to 2023. The vegetation parameters include biomass (BM), crop yield (CY), leaf area index (LAI), biodiversity (BD), pigment content (PC), nitrogen content (NC), water content (WC), net primary productivity (NPP), gross primary productivity (GPP), plant height (PH), photosynthetically active radiation (PAR), fraction of photosynthetically active radiation (FPAR), fractional vegetation cover (FVC), clumping index (CI), solar-induced chlorophyll fluorescence (SIF), and vegetation optical depth (VOD). Solid lines in light blue, pink, and black indicate the trend in the number of publications with hyperspectral data, deep learning methods, and integration of hyperspectral data with deep learning techniques for the remote sensing inversion of vegetation parameters, respectively. (B) The coefficient of determination (R^2^) and relative root-mean-square error (rRMSE) were used for statistical analysis of the accuracy in inverting wheat biophysical and biochemical parameters using four methodologies—statistical models, physical models, machine learning, and deep learning—along with hyperspectral (HS) or multispectral (MS) data.

Based on the Web of Science Core Collection, we collected over 40,000 papers published since 1990 with the topic of remote sensing inversion of vegetation parameters. CiteSpace software was subsequently applied for cluster analysis to identify key vegetation parameters, which were then cross validated against previous authoritative studies. A targeted search within the Web of Science Core Collection was further conducted to locate relevant articles specifically addressing these parameters. The retrieved articles were categorized by vegetation parameters and research topics and their numbers summarized (Figure 1A). Given the extensive volume of literature retrieved, ChatGPT was employed to assist in efficiently analyzing and summarizing the abstracts. This process enabled systematic classification and analysis of the papers based on their methodologies, conclusions, and contributions. Finally, an in-depth analysis of the advancements in remote sensing inversion methods for vegetation parameters was performed.

Since 1990, the remotely sensed inversion of biophysical parameters, such as biomass (BM), crop yield (CY), leaf area index (LAI), and plant height (PH), along with biochemical parameters such as nitrogen content (NC), pigment content (PC), and water content (WC), has been widely studied and accounts for over 70% of total research. Specifically, BM, CY, and LAI parameter inversion studies represent 16.7%, 14.6%, and 10.3%, respectively, while NC, PC, and WC studies account for 9.8%, 8.4%, and 7.7%, respectively. In contrast, parameters such as fractional vegetation cover (FVC), clumping index (CI), photosynthetically active radiation (PAR), fraction of photosynthetically active radiation (FPAR), vegetation optical depth (VOD), net primary productivity (NPP), gross primary productivity (GPP), and biodiversity (BD) have been less studied, collectively accounting for less than 30%. Notably, research on BD exceeds 10% and appears to be increasing at a high rate.

Particular attention was devoted to emerging trends in hyperspectral remote sensing technology applications and the development of deep learning approaches.^2^ We found that since 1996, hyperspectral remote sensing technologies have become important means for monitoring changes in plant growth. The number of papers on the inversion of vegetation parameters based on this technology has been steadily increasing at a compound annual growth rate (CAGR) of 13.57%. Since 2016, deep learning methods have also gradually become important tools for the inversion of vegetation parameters, with a CAGR of 47.2% for related research. In recent years, the integration of hyperspectral remote sensing and deep learning for vegetation parameter retrieval has gained traction, with a CAGR of 28.5% for related research.

The wheat biophysical parameters (BM, CY, and LAI) and biochemical parameters (NC, PC, and WC) were selected as examples to analyze their remote sensing inversion accuracy through various methods, including statistical models, physical models, machine learning, and deep learning (Figure 1B). The accuracy metrics selected for our analysis were coefficient of determination (R^2^) and relative root-mean-square error (rRMSE). The R^2^ values were obtained directly from the data provided in the papers, and the rRMSE values were derived from the RMSE values reported in the paper by dividing the mean of the corresponding dataset. We found that the inversion of these vegetation parameters using hyperspectral data has an accuracy that is 20% higher than that using multispectral data that have only a few bands. This improved performance can be partly attributed to hyperspectral data’s ability to enable more precise vegetation classification, which, in turn, enhances the accuracy of vegetation parameter inversion. Compared to other methods, deep learning showed higher accuracies in wheat inversion retrievals of morphological features and growth-related vegetation parameters, with an average R^2^ of about 0.87 and an average rRMSE of about 14%, where R^2^ indicates the inversion method’s goodness of fit to the training data, while rRMSE measures the inversion method’s prediction accuracy. However, deep learning methods were unable to improve biochemical parameter inversion accuracy compared with traditional methods. This was likely due to the direct adoption of current deep learning models, such as recurrent neural networks and convolutional neural networks, from the fields of natural language processing and computer vision into remote sensing applications. These models are primarily designed to extract temporal, structural, and textural information rather than the spectral information that is crucial in biochemical remote sensing analysis.

Deep learning models are distinguished by their complexity, involving a large number of parameters that require training with massive input sample datasets. This stands in sharp contrast to the limited availability of field samples in vegetation studies. The accurate estimation of vegetation biochemical parameters is highly dependent on detailed spectral information; however, previous research has shown that the number of field measurement samples used to train deep learning models for this purpose is often critically inadequate. Most studies utilize only a few dozen to a few hundred samples, falling far short of the extensive data requirements necessary for optimal deep learning model performance.^3^ Currently, the inversion of vegetation biochemical parameters primarily relies on the use of hyperspectral vegetation indices combined with machine learning models. This is because machine learning models with manually designed feature patterns require smaller datasets compared to their deep learning counterparts.^3^ To overcome the limitations of insufficient sample size in training deep learning models, recent research studies have employed small sample transfer learning techniques, which involve the pre-training of a model on a large-scale simulated dataset and then fine-tuning it on smaller samples of actual field-measured data.^4^^,^^5^ Since simulated data can be generated quickly and can encompass a variety of environmental conditions that are not easily accessible in real-world data, it can be a cost-effective alternative to collecting and labeling real-world data compared to traditional methods.^4^ This physically constrained deep learning model has been shown to significantly improve the accuracy in retrievals of vegetation biochemical and biophysical parameters compared with pure data-driven and radiative transfer model inversion approaches.^4^^,^^5^ Furthermore, it enhances the efficiency and generalization ability in parameter inversions, thereby providing a more robust and reliable approach to vegetation parameter retrievals.

The emergence of foundational models (FMs) is likely to significantly impact vegetation remote sensing. These models offer two key advantages: (1) they are trained on vast amounts of unlabeled data using self-supervised learning, allowing them to extract robust and widely applicable generalizable features, and (2) these pre-trained models can then be easily adapted to specific tasks in vegetation remote sensing with the help of zero-shot learning and few-shot learning, even with limited or no additional training data.^4^ This capability ensures that an FM trained with simulated data generated by physical models can address the issue of insufficient real-world sample sizes. The transformer architecture, which forms the core of most current FMs,^4^ has demonstrated exceptional ability in extracting features from sequential data.^2^ This capability makes it particularly promising for mining spectral features in remote sensing applications. By carefully designing the model structure and coding strategy, we can significantly improve the efficiency of spectral information utilization and feature extraction, thereby fully leveraging the advantages of hyperspectral data. Finally, FMs also possess the capability to integrate multimodal data,^2^^,^^4^ enabling the mining of spectral features while complementing other feature-related vegetation parameters, such as spatial texture and phenological characteristics, thereby enhancing both the accuracy and efficiency of FMs in vegetation parameter inversion tasks.

In summary, the integration of hyperspectral remote sensing data with deep learning holds significant potential for the inversion of vegetation parameters. The challenge lies in leveraging deep learning techniques to enhance the extraction of spectral features and the accuracy of vegetation biophysical and biochemical parameter inversion as well as in developing end-to-end deep learning models that achieve a balance between efficiency, accuracy, and robustness. This convergence of advanced deep learning techniques and remote sensing data signals a transformative era in vegetation parameter estimation that promises unprecedented accuracy and efficiency in ecosystem monitoring and applications.

## Funding And Acknowledgments

This work was supported by the 10.13039/501100001809National Natural Science Foundation of China (42030111 and 42471372).

## Declaration of interests

The authors declare no competing interests.
